# Multi-Camera Digital Image Correlation in Deformation Measurement of Civil Components with Large Slenderness Ratio and Large Curvature

**DOI:** 10.3390/ma15186281

**Published:** 2022-09-09

**Authors:** Yuntong Dai, Hongmin Li

**Affiliations:** 1 College of Civil Engineering, Anhui Jianzhu University, Hefei 230601, China; 2 College of Materials Science and Engineering, Nanjing Forestry University, Nanjing 210037, China

**Keywords:** multi-camera digital image correlation, continuous-view, deformation measurement, large slenderness ratio, large curvature, civil component

## Abstract

To address the limitations of conventional stereo-digital image correlation (DIC) on measuring complex objects, a continuous-view multi-camera DIC (MC-DIC) system and its two forms of camera arrangement are introduced. Multiple cameras with certain overlapping field of view are calibrated simultaneously to form an overall system for measuring the continuous full-surface deformation. The bending experiment of coral aggregate concrete beam and the axial compression experiment of timber column are conducted to verify the capability of continuous-view MC-DIC in deformation measurement of civil components with large slenderness ratio and large curvature, respectively. The obtained deformation data maintain good consistency with the displacement transducer and strain gauge. Results indicate that the continuous-view MC-DIC is a reliable 3D full-field measurement approach in civil measurements.

## 1. Introduction

Digital image correlation (DIC) has been developed into a widely applied deformation measurement technique, with the advantages of relatively simple setup and non-contact, non-destructive, full-field measurement [1,2]. Binocular stereo-vision [3,4] in conjunction with DIC leads to three-dimensional (3D) DIC, also known as stereo-DIC [5]. Dual cameras are placed to simultaneously capture image sequences of object surface from different view angles, and the full-field 3D surface topography and deformation are estimated through tracking the random distribution of natural texture or artificial speckles on surface. Substantial work has been done to improve the measurement accuracy and efficiency [6,7,8,9,10,11,12], and extend the application range [13,14,15,16,17,18,19,20] of stereo-DIC.

The effective field of view (FOV) covering the measured surface is the necessary condition to complete the full-surface deformation measurement. The conventional stereo-DIC with dual cameras has some limitations and drawbacks in the following cases. For complex object with large curvature, especially the cylinder, the entire surface is beyond the FOV of dual-camera system, resulting in an incomplete or failed measurement. To address the limitations, the multi-camera DIC (MC-DIC) technique [21,22,23], combining stereo-DIC with the multi-camera synchronous acquisition system, has come into being. The high-precision 3D deformation measurement of objects with large curvature could be realized. Alternatively, for the dual-camera measurement of object with large slenderness ratio, it is often necessary to adjust the working distance of cameras or change lenses to provide enough FOV for complete cover of the entire surface. These adjustments will cause a significant waste of the camera resolution in the shorter direction. In this regard, MC-DIC is a sound solution to improve the measurement accuracy on the basis of making full use of the camera resolution. Moreover, MC-DIC is also an effective approach to improve the deformation measurement accuracy of common objects. Typically, the measurement accuracy of stereo-DIC can be improved by both the algorithm and hardware. The improvement of camera signal-to-noise ratio can reduce noise, thereby obtaining higher displacement identification accuracy. Moreover, the increase of camera resolution can increase the gauge length of strain calculation, thereby improving the strain measurement accuracy. However, these rely on hardware advancement, which is a long process. MC-DIC does not rely on a large resolution increase of each single camera, but effectively improves the spatial resolution by increasing the number of current cameras, so that the measurement accuracy of DIC is improved directly.

Several MC-DIC systems with different camera configurations have been proposed in the literature, which can be mainly categorized into discrete-view MC-DIC system [24,25,26,27,28,29,30] and continuous-view MC-DIC system [21,22,31,32,33,34], depending on whether there are overlapping FOVs. Notably, the unification of local and global coordinate systems is a crucial process for MC-DIC measurement. The calibration approaches involved in the above two systems are a combination of planar calibration method and other auxiliary techniques, and planar calibration method only, respectively.

The multiple cameras in discrete-view MC-DIC system have no overlap on FOVs. For instance, Malesa [24,25] and Malowany [26] presented a spatial data stitching algorithm including camera calibration and geometrical transformation, among which the positions of fiducial markers are determined by stereo-DIC units and geodetic surveying or laser tracker. The proposed MC-DIC system with distributed, non-overlapping FOV was adopted the complete in-situ measurement of large engineering construction. Dong et al. [27,28] developed a target-based calibration method and a data merging algorithm for MC-DIC system, which can be applied in separated FOVs. Combined with close-range photogrammetry, the spatial positions of identification points are determined to calibrate the extrinsic parameters of cameras, so as to convert all measurement results to the same coordinate system. Besides, another strategy for discrete-view MC-DIC is that multiple sets of stereo-DIC subsystem are used to measure different portions of the whole object surface to obtain the discrete full-surface deformation. For instance, Chen [29] utilized four stereo-DIC subsystems composed of eight circumferentially oriented cameras to monitor the distributed 360° full-circle deformation of concrete cylinders. Zhao [30] proposed a global-local MC-DIC method especially for stress concentration problems, in which the whole area and the specific local area were covered separately by two different DIC subsystems. In general, discrete system is incapable of achieving continuous measurement over the whole object surface due to the distributed FOVs.

Whereas, for the continuous-view MC-DIC system, a certain overlap of adjacent FOVs is required. Multiple cameras are unified into one global coordinate system to form an overall measurement system, which can obtain the continuous full-surface deformation. Thus, the full-circle 360° measurement of cylinder is theoretically possible. Orteu [21] and Harvent [22] developed a simultaneous multi-camera calibration procedure using multiple-image triangulation and bundle adjustment. All the calibration views are required to be visible by the specified master camera, so as to convert individual data into a common coordinate system. The proposed MC-DIC system was used to measure the 3D shape and deformation of a sheet metal part during the forming process and an aeronautic part, successively. Wang et al. [31] proposed a MC-DIC set-up composed of two conventional stereo-DIC subsystems to measure a sheet-metal specimen subjected to an Erichsen test. The calibration of each subsystem is conducted separately, and then through frame transformations, the global point clouds are formed by integrating the geometries and displacements from different individual subsystems. Chen et al. [32,33] developed a MC-DIC method based on cluster approach to measure the entire contour, displacement and strain field of a piece of sheet alloy loaded with infrared heaters. Through pre-calibration, the two stereo-DIC pairs with a 10% overlapping FOV are linked together as one single system to evaluate the full-surface deformation. Hwang et al. [34] proposed a MC-DIC system with a movable frame which can provide different overlapping FOVs and view angles flexibly. The three-camera MC-DIC system with semi-circular configuration was utilized to reconstruct the 3D surface of a cracked cylinder.

So far, MC-DIC has some precedents in various domains, such as material testing, automotive and aerospace industries, biomedicine, etc. Johanson et al. [35] applied the MC-DIC system to simultaneously view the strain fields from two opposing surfaces of tensile specimen for failure analysis. Li et al. [36,37] used the MC-DIC system to measure the whole-field thickness strain and determine the true stress–strain curve, including the post-necking strain of a tensile specimen, respectively. Poozesh et al. [38,39] proposed a MC-DIC system with certain overlapped FOV for measuring a utility-scale wind turbine blade. Gardner et al. [40] applied the MC-DIC system to measure the buckling response of the launch-vehicle-like cylindrical shell. Solav et al. [41] put forward a framework for the in-vivo shape and deformation measurement of residual limbs using the MC-DIC system. Tong et al. [42] utilized the MC-DIC system to measure the 3D profile and deformation of human arm skin during arm wrestling. Wei et al. [43] proposed a MC-DIC system with four high-speed cameras for the 3D full-field measurement of a multi-layer structure model in a large shaking table.

The application of the continuous-view MC-DIC system in civil measurements will be the main focus of the present paper. The most common civil components, namely beam as flexural member and column as vertical load-bearing member, will be the research objects. The aim of this study is not only to complete the continuous full-surface measurement of civil components with large slenderness ratio and large curvature, but also to broaden the measurement range and improve the measurement accuracy of DIC in civil measurements.

The remainder of this paper is organized as follows. Section 2 briefly introduces the fundamental principles, especially the camera calibration, the measurement uncertainty, and the two forms of camera arrangement for the continuous-view MC-DIC system. In Section 3, the bending experiment of a coral aggregate concrete beam is taken as an example to demonstrate the validity of continuous-view MC-DIC in the deformation measurement of civil component with large slenderness ratio. In Section 4, the axial compression experiment of timber column is conducted to verify the capability of continuous-view MC-DIC in the deformation measurement of civil components with large curvature. Finally, the conclusions are summarized in Section 5.

## 2. Fundamental Principles

### 2.1. Principle of Stereo-DIC

**Figure 1:**
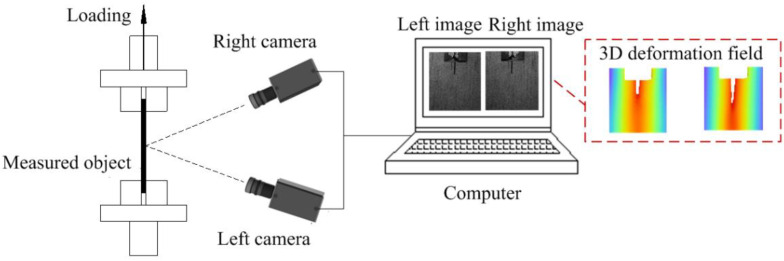
Experimental setup of the conventional stereo-DIC method.

**Figure 2:**
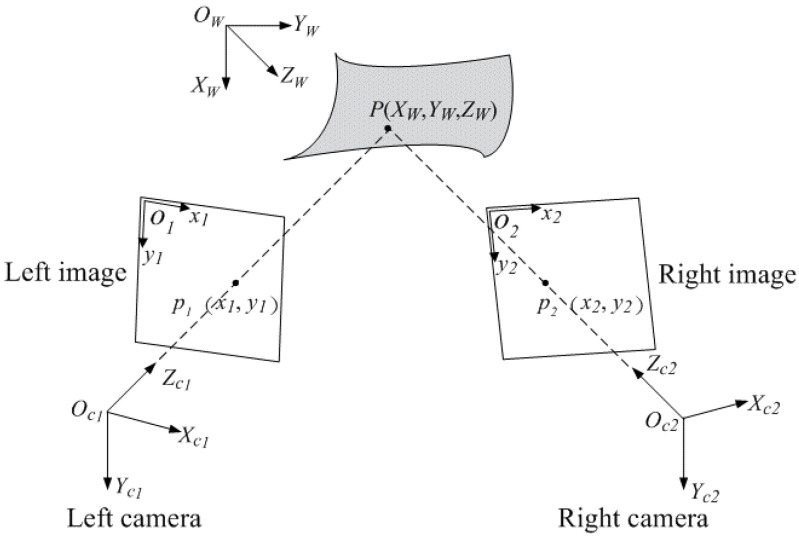
Principle of binocular stereo-vision.

Figure 1 presents the experimental setup of the conventional stereo-DIC method, which combines DIC with the principle of binocular stereo-vision (as shown in Figure 2). At least two synchronized cameras are used to observe the same area from different view angles, and then spatial and temporal image correlation are performed to obtain the 3D shape and deformation. The basis of DIC is the correlation operation, among which the correlation function [5] is applied to evaluate the similarity of grey level in subsets before and after deformation, and the strain field is calculated from the sub-pixel displacement field obtained by correlation matching. Meanwhile, the subset shape function [8] is used to approximately describe the position and shape of the deformed subset, including the zero-order shape function for rigid body translation, the first-order shape function for uniform deformation, such as rigid body rotation, tension and compression deformation, and the second-order shape function considering the strain gradients for non-uniform deformation. Furthermore, the sub-pixel search directly determines the measurement accuracy of DIC. The typical algorithms include the Newton–Raphson (NR) algorithm [9] and inverse compositional Gauss–Newton (IC-GN) algorithm [11]. The random, high-contrast speckle patterns [12] are the carrier of deformation for DIC estimation. Commonly used speckle fabrication methods include spray painting, airbrush gun, lithography, screen printing, thermal transfer printing, water transfer printing, and so on.

To implement the stereo-DIC measurement, the camera calibration is an essential step for determining the correspondence between the 3D space point and the two-dimensional (2D) image points. The basic principle of binocular stereo-vision [3,4] is presented in Figure 2. $P(X_{W},Y_{W},Z_{W})$ is a 3D physical point on the measured surface, $p_{1}(x_{1},y_{1})$ and $p_{2}(x_{2},y_{2})$ represent its stereo projections in the images of left and right cameras. By triangulation, the 3D coordinates of the physical point *P* in the world coordinate frame can be recovered from the two image points $p_{1}$ and $p_{2}$ according to the following equation:

(1) $$s_{i}\left[\begin{array}{c} x_{i}\\ y_{i}\\ 1 \end{array}\right]=M_{i}\left[\begin{array}{c} X_{W}\\ Y_{W}\\ Z_{W}\\ 1 \end{array}\right]=A_{i}\left[\begin{array}{cc} R_{i} & t_{i}\\ 0 & 1 \end{array}\right]\left[\begin{array}{c} X_{W}\\ Y_{W}\\ Z_{W}\\ 1 \end{array}\right]\begin{array}{cc} , & i=1,2 \end{array}$$

where $s_{i}$ denotes the scale factor, $M_{i}$ represents the projection matrix obtained by the multiplication of intrinsic parameters matrix $A_{i}$ and extrinsic parameters $\left[R_{i},t_{i}\right]$ with $R_{i}$ being a 3 × 3 rotation matrix and $t_{i}$ being a 3 × 1 translation vector, respectively. The intrinsic parameters (i.e., principal point coordinates $c_{x},c_{y}$, effective focal length $f_{x},f_{y}$, skew factor γ, etc.) are the inherent camera properties. Further, the extrinsic parameters indicate the 3D position and orientation of cameras with respect to the word coordinate frame.

Then, through temporal matching, the 3D displacement of the physical point *P* can be computed by subtracting the 3D coordinates of the point at two instants. The 3D strain can be estimated from the displacement field using the local least-square fitting method [44], which is suitable for both uniform and non-uniform deformation.

### 2.2. Principle of Continuous-View MC-DIC

**Figure 3:**
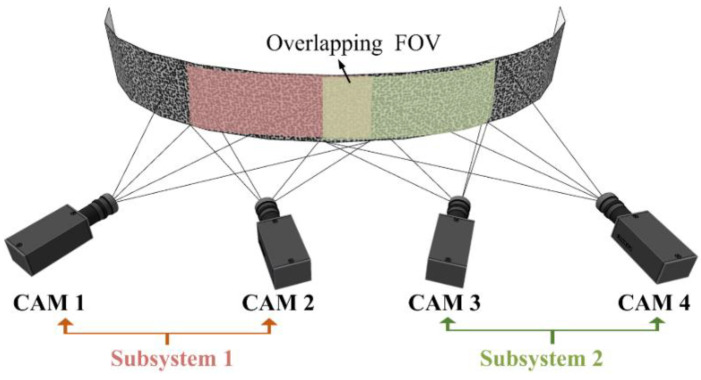
Schematic of a continuous-view MC-DIC system containing two stereo-DIC subsystems composed of four cameras: red shaded area and green shaded area denote the FOV of subsystem 1 and subsystem 2, respectively; yellow shaded area in middle is the overlapping FOV between subsystem 1 and 2.

In the continuous-view MC-DIC system, multiple cameras are used to simultaneously monitor local portions of the measured surface, among which each local portion is measured based on the binocular stereo-vision principle. Schematic of a continuous-view MC-DIC system containing four cameras is illustrated in Figure 3. To realize the continuous full-surface measurement, the following three points need to be noted. Firstly, each local portion should be covered by the FOV of at least one set of stereo-DIC subsystem, so that each point on the measured surface can be successfully reconstructed. Secondly, the adjacent local portions should fulfill certain requirements regarding FOV overlap to ensure the integrity of continuous deformation measurement. Thirdly, the planar calibration target is utilized as the conversion medium between the local and global coordinate systems. Thus, all data points are unified to form one overall system for 3D deformation estimation.

For multi-camera measurement, the first and most critical step is camera calibration. As described in previous subsection, camera calibration is the procedure which consists in determining the intrinsic and extrinsic parameters of cameras. Enough 3D points with known coordinates are required to estimate those parameters. Generally, on basis of the well-established stereo-vision calibration method [45], the planar calibration target with spaced black and white grids or circular dots is selected to complete the camera calibration.

Without loss of generality, we suppose that the continuous-view MC-DIC system is composed of *N* cameras, each camera captures *n* calibration images of the calibration target at different positions and orientations, and the calibration target contains *m* feature points. For simplicity, special marks are added on the calibration target to predefine the *X*, *Y* coordinate axes and origin point *O* of the world coordinate frame, and the normal direction of plane *XOY* is defined as the *Z* coordinate axis. Therefore, the transformation from the 3D space point $P_{j}$ in the world coordinate frame to the 2D image point in the image coordinate frame in pixel can be described by

(2) $$f\left(P_{j},A_{k},R_{ik},t_{ik}\right)$$

with subscript *i* (*i* = 1, 2, …, *n*) denoting the different positions and orientations of calibration target, subscript *j* (*j* = 1, 2, …, *m*) representing the different feature points on calibration target, and subscript *k* (*k* = 1, 2, …, *N*) indicating the different cameras. For each camera, the intrinsic parameter matrix $A_{k}$ is constant. Besides, the extrinsic parameters $\left[R_{ik},t_{ik}\right]$ are changeable during the calibration process, which indicates the relationship between *k*th camera and calibration target in *i*th position and orientation.

The residual function, namely 2D Euclidean distance between the estimated and actual projection, is presented as follows

(3) $$\sum_{i=1}^{n}\sum_{j=1}^{m}\sum_{k=1}^{N}{\left\|p_{ik}^{j}-f\left(P_{j},A_{k},R_{ik},t_{ik}\right)\right\|}^{2}$$

Where $p_{ik}^{j}$ represents *j*th feature point on the calibration target extracted in *i*th calibration image captured by *k*th camera, and $f\left(P_{j},A_{k},R_{ik},t_{ik}\right)$ represents the estimated projection of the *j*th point $P_{j}$ onto *i*th calibration image captured by *k*th camera through triangulation.

When this residual function takes the minimum value, it means that the current camera parameters are the optimal solution. The initial guess of above camera parameters can be firstly computed by linear solution, and then the exact value will be obtained by nonlinear optimization process. For instance, the sparse bundle adjustment method [46] based on the Levenberg–Marquardt algorithm can be used to perform the overall optimization iteration on the camera parameters and 3D coordinates.

After multiple cameras are calibrated at the same time to become a whole measurement system, the spatial and temporal image correlation are conducted successively. The spatial subset matching in images taken by different cameras at the same instants will complete the 3D reconstruction at current instant. Then, combined with the temporal subset matching in images taken by the same camera at different instants, the 3D displacement can be estimated. Afterwards, the 3D strain will be calculated from the displacement.

### 2.3. Measurement Uncertainty of MC-DIC Compared to Stereo-DIC with Dual Cameras

**Figure 4:**
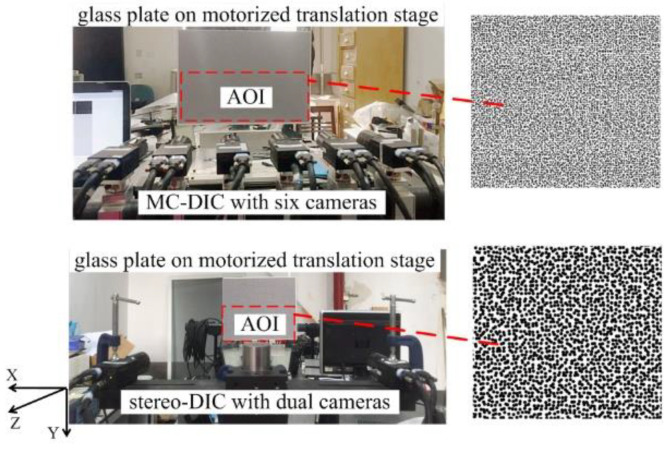
Rigid motion test of a glass plate.

To assess the measurement uncertainty of MC-DIC, a rigid motion test is conducted. As depicted in Figure 4, a glass plate is fixed on the high-precision motorized translation stage, which can provide movement in a certain direction. One side of the plate is monitored by a continuous-view MC-DIC system with six cameras, and the other side is a conventional stereo-DIC system with dual cameras. A known rigid motion of the plate is given by the translation stage, being simultaneously measured by the two systems. Both systems are composed of the same industrial cameras GRAS-50S5M-C (Point Grey Research Inc., Richmond, BC, Canada) with resolution of 2448 × 2048 pixels and pixel size of 3.45 μm and the same Kowa lenses with focal length of 25 mm. The measuring Area of Interest (AOI) of two systems is both 400 mm × 160 mm. To avoid the influence of different speckle sizes, speckles with suitable sizes are generated by the computer according to the FOV difference of single camera in the two systems, and then pasted on both sides of the plate after printing.

**Figure 5:**
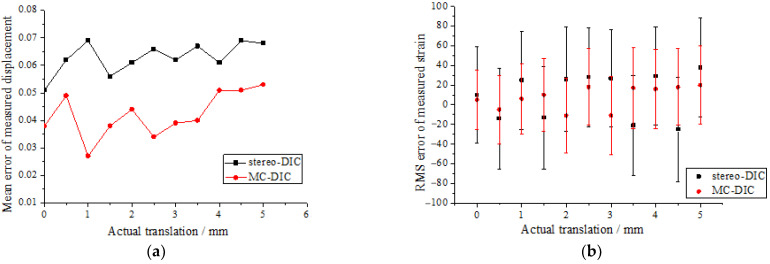
(**a**) Mean errors of the measured displacements by two systems; (**b**) RMS errors of the measured strains by two systems.

The plate is continuously translated 10 times with the speckle images being captured. The translation quantity of two adjacent images is 0.5 mm, and the maximum translation displacement is 5 mm. Figure 5a presents the mean errors of the measured displacements by two systems. MC-DIC is capable of improving the displacement measurement accuracy to some extent. Furthermore, given that the strain field of rigid body motion is theoretically equal to zero, the measured strain represents the measurement error. It can be seen from Figure 5b that the root-mean-square (RMS) error of strain measured by MC-DIC is within ±60 με, and that by stereo-DIC is within ±95 με. The strain measurement accuracy of the MC-DIC system is also improved compared to the stereo-DIC system.

### 2.4. Camera Arrangements for Continuous-View MC-DIC

Summarized from the existing studies, two configurations of MC-DIC system according to different applications are mainly considered, that is the surrounding cameras for cylindrical object and the wall-of-cameras for large object. It is worth noting that the present section will discuss camera arrangements of continuous-view MC-DIC measurement for objects with large slenderness ratio and with large curvature, from the perspective of overlapping FOV. Due to the fact that the cylindrical surface can be unfolded somewhere to obtain an elongated plane, only the elongated planar surface is used for schematic display of camera arrangements. The differences between the circumferential arrangement for object with large curvature and the parallel arrangement for object with large slenderness ratio will be pointed out.

**Figure 6:**
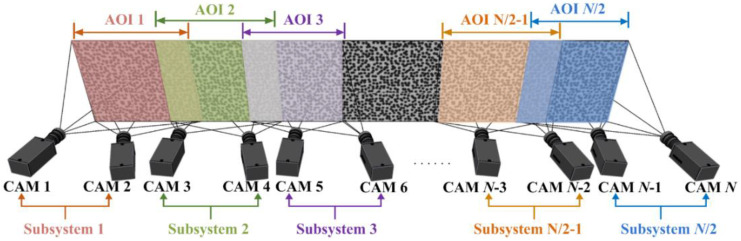
First form of camera arrangement for continuous-view MC-DIC system.

The first form of camera arrangement is presented in Figure 6. Two cameras composed of one set of stereo-DIC subsystem and multiple sets of stereo-DIC subsystem are arranged parallel to or circumferentially around the object to measure the local portions of entire surface. A certain overlapping FOV between adjacent stereo-DIC subsystems is required to guarantee the continuous deformation measurement.

Take the parallel arrangement for elongated planar surface as an example, each set of subsystem observes the corresponding AOI, and a certain AOI overlap exists between the neighboring subsystems. Specifically, cameras 1 and 2 constitute subsystem 1 for measuring AOI 1, cameras 3 and 4 constitute subsystem 2 for measuring AOI 2, and cameras 5 and 6 constitute subsystem 3 for measuring AOI 3. There is a partial overlap between AOI 1 and AOI 2, as well as that between AOI 2 and AOI 3. The intermediate cameras are also arranged in the same manner. The penultimate subsystem *N*/2-1 composed of cameras *N*-3 and *N*-2 measures AOI *N*/2-1, the last subsystem *N*/2 composed of cameras *N*-1 and *N* measures AOI *N*/2, and AOI *N*/2-1 and AOI *N*/2 has certain overlap. In addition, for object with large curvature, especially the cylinder, multiple sets of stereo-DIC subsystem are arranged circumferentially around the object. The camera arrangement in Figure 4 can also be adopted with minor adjustment, namely, the last subsystem *N*/2 must have a partial AOI overlap with the first subsystem 1, so as to guarantee the continuous full-circle measurement of cylinder.

**Figure 7:**
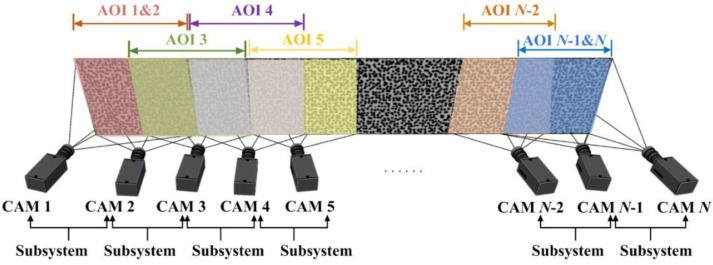
Second form of camera arrangement for continuous-view MC-DIC system.

Figure 7 describes the second form of camera arrangement, multiple cameras are arranged parallel to or circumferentially around the object, with every two adjacent cameras comprising a stereo-DIC subsystem. To complete the continuous full-surface measurement, at least half AOI overlap between every two adjacent cameras is required, so that each measurement point on object surface is ensured to be captured by at least two cameras.

Similarly, the parallel arrangement for object with large slenderness ratio is illustrated for instance. The AOIs of cameras 1 and 2 are completely coincident, forming a stereo-DIC subsystem to measure AOI 1&2. The same goes for the two cameras at the end, the AOIs of cameras *N*-1 and *N* exhibit a complete overlap to constitute a stereo-DIC subsystem for measuring AOI *N*-1& *N*. For cameras in the middle, the AOIs of adjacent cameras overlap by more than half, and subsystems are composed of every two neighboring cameras.

**Figure 8:**
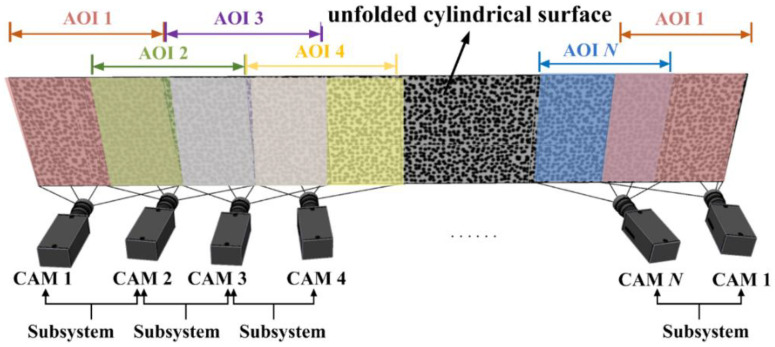
Second form of camera arrangement especially for cylinder.

Moreover, for object with large curvature, especially the cylinder, we can utilize the revised edition of the second arrangement, as shown in Figure 8, to place cameras circumferentially around the object. The two cameras at each end do not need complete overlapping AOI, but camera 1 and camera *N* should be linked together with an AOI overlap more than half. To achieve continuous full-circle measurement of cylinder, the AOIs of every two neighboring cameras overlap by more than half, especially camera 1 and camera *N*, and subsystems are composed of every two adjacent cameras having overlap AOI.

## 3. Bending Experiment of Coral Aggregate Concrete Beam

In this section, the bending experiment of coral aggregate concrete beam is employed to demonstrate the effectiveness of continuous-view MC-DIC in deformation measurement of civil component with large slenderness ratio. The deflection distribution, deformation characteristics, and crack propagation on beam surface are obtained, and the MC-DIC result is compared with the displacement transducer.

### 3.1. Experimental Program

Coral aggregate concrete is prepared from coral reef (coral stone as coarse aggregate and coral sand as fine aggregate), cement, admixture and seawater in a certain proportion [47]. Basalt Fiber Reinforced Plastics (BFRP) bars can be used to replace steel bars as structural reinforcements for their advantages of light weight, high strength, good acid and alkali resistance and ability to improve structural durability in extreme corrosive environments [48]. Therefore, using coral aggregate concrete instead of ordinary concrete and BFRP bars instead of steel bars can reduce construction costs and improve economic benefits while achieving high durability and long service life of building structures [49].

The tested specimens are divided into reinforced coral aggregate concrete beam and BFRP-reinforced coral aggregate concrete beam with the same size. The two experimental results are compared to study the flexural performance of coral aggregate concrete beam, including the bearing capacity, failure mode, deflection distribution and crack development. Note that only one group of specimens is chosen as an example to demonstrate the effectiveness of continuous-view MC-DIC in the deformation measurement of civil components with large slenderness ratio in this section.

**Figure 9:**
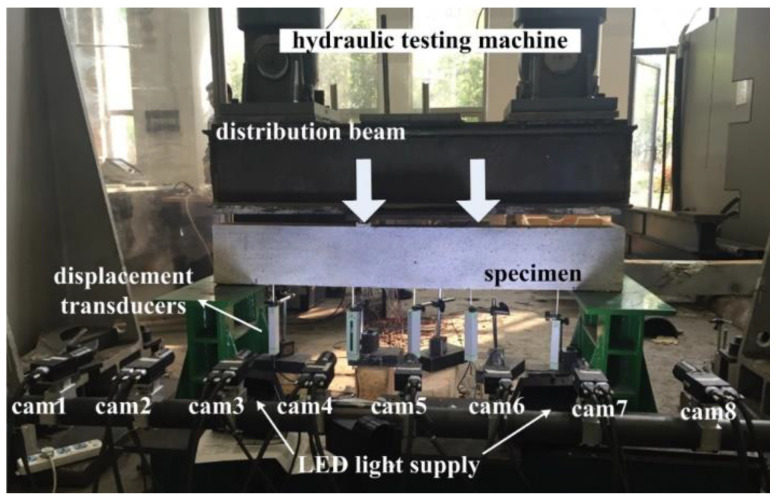
Experimental setup for the bending experiment of coral aggregate concrete beam.

The cross-section size and length of example beam are 200 mm × 120 mm and 1350 mm, respectively. Random black-and-white speckles are produced by spray painting. The hydraulic testing machine is used for loading, and the four-point bending is realized with a distribution beam. A hybrid control loading strategy of load control first and then displacement control is adopted. Before the yielding of steel bar or BFRP bar, the loading level is set to 5 kN per stage, and after the bar has yielded, displacement control loading with the mid-span deflection difference of 0.5 mm is conducted until the specimen failure, so as to prevent the possible damage of measuring instruments caused by the concrete crushing and spalling. The experimental setup is shown in Figure 9, including eight industrial cameras (Point Grey GRAS-50S5M-C) with resolution of 2448 × 2048 pixels and pixel size of 3.45 μm, eight Kowa lenses with focal length of 25 mm, and LED light supplies.

The system construction process is as follows: (1) Considering the specimen size and the expected deformation, the total measurement area of MC-DIC is set to be 1400 mm × 300 mm, and eight cameras are arranged in parallel along the beam length. (2) After determining the relative distance between cameras and specimen, eight cameras are evenly arranged at a distance of 200 mm with FOV of about 400 mm × 400 mm for each camera. The FOVs of cameras 1 and 2 are completely coincident, the FOVs of adjacent cameras between camera 2 and camera 7 overlap by at least 50%, and the FOVs of cameras 7 and 8 are also completely coincident. (3) Eight cameras are mounted rigidly to the high-strength carbon fiber tube through sleeves, and then the tube is fixed on two tripods at the two ends, forming a stable overall system. During the experiment, the exposure time of each camera is fixed to 25 ms and the moderate aperture of each lens is set. For the given aperture and exposure time, through the adjustment of LCE light supplies, sufficient contrast can be obtained while limiting the motion blur. Eight cameras are synchronously controlled to capture images with acquisition frame rate of 1 frame/s. Simultaneously, displacement transducers are installed for the comparison between optical and electrical measurements.

### 3.2. DIC Results and Discussion

**Figure 10:**
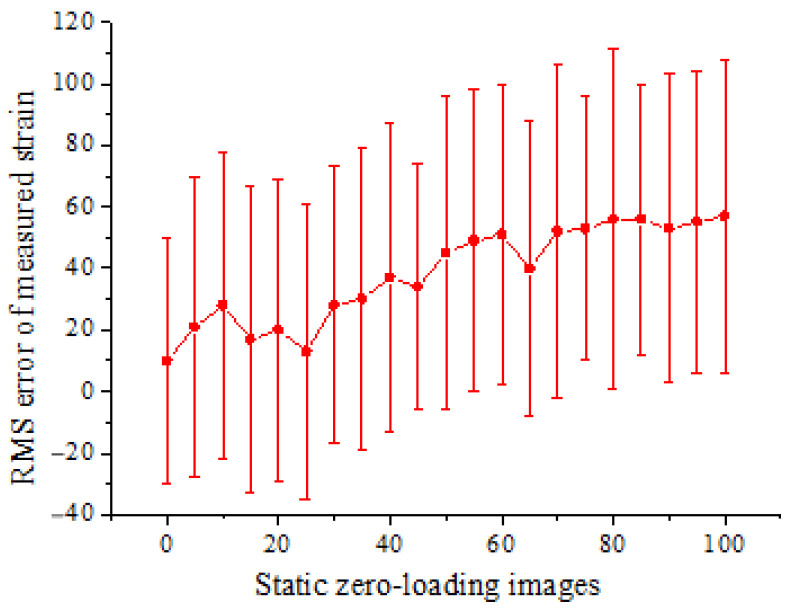
RMS error of strain computed from the static undeformed beam.

In order to quantify the noise or error, a series of static images are acquired from the undeformed specimen before loading. The RMS error of strain computed from the static zero-loading images is shown in Figure 10. The measured strain is within ±110 με, showing an overall slight increasing trend.

**Figure 11:**
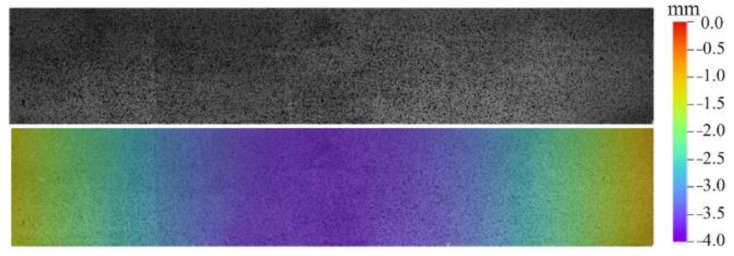
Full-surface speckle pattern and contour of the beam surface.

Through adjusting DIC calculation parameters both in noise analysis and actual experiment analysis, the parameter selection can be confirmed considering the balance between noise filtering and accuracy. The subset size is set to 35 × 35 pixels, the grid step size is set to 7 pixels, and the strain calculation window size is set to 15 × 15 calculation points. The full-surface speckle pattern and the 3D surface topography are presented in Figure 11. The figure indicates that the continuous-view MC-DIC system can completely restore the speckle image and 3D contour of the entire beam with large slenderness ratio.

**Figure 12:**
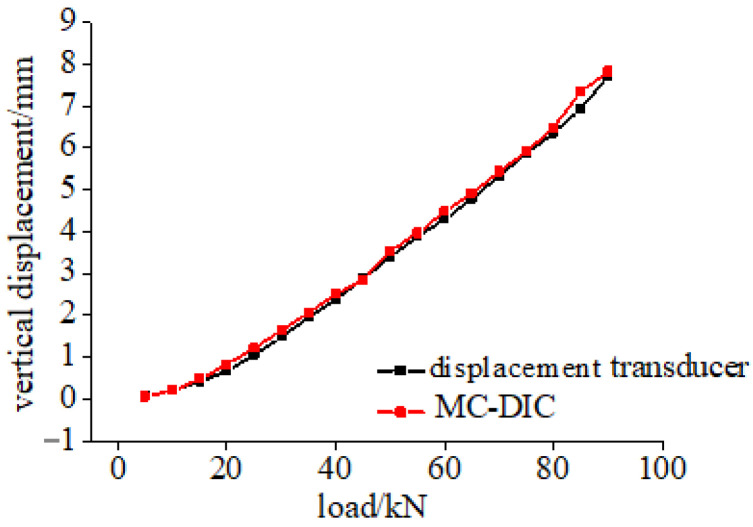
Results comparison of MC-DIC and displacement transducer.

Take the bottom mid-span point as an example, the displacement calculation result of MC-DIC is compared with the measurement data of displacement transducer. Clearly observed from Figure 12, the two curves are basically consistent, and the relative error is within 3%. The main reason for this difference is that there is friction between the measuring rod and sleeve of displacement transducer, when the deformation is too small, the effective sliding of the measuring rod is difficult, resulting in a smaller value of the displacement transducer. It should also be noted that a certain deviation exists between the positions of DIC calculating point and actual measuring point of displacement transducer, due to the manual extraction of DIC data point.

**Figure 13:**
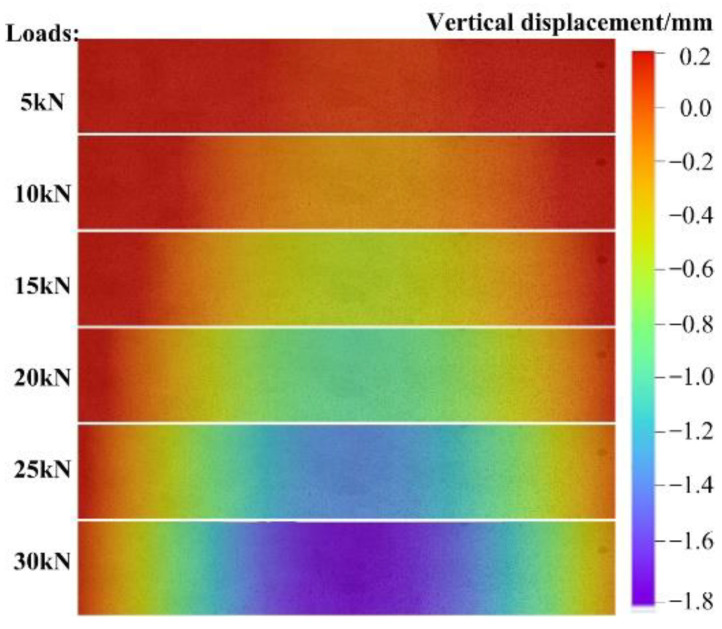
Vertical displacement evolution on beam surface under different loads.

Figure 13 intuitively reveals the full-field displacement evolution in vertical direction. The displacement increases gradually with the increase of load, and the maximum displacement occurs at the mid-span position. This result is consistent with the deformation characteristics of the four-point bending beam. Moreover, the displacement field vividly shows the process of deformation gradually spreading from the mid-span to the supports.

**Figure 14:**
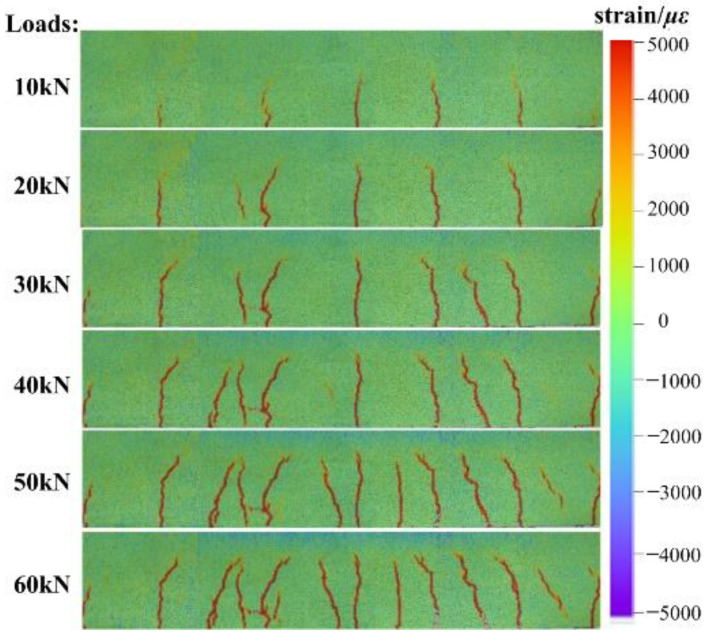
Crack propagation on beam surface under different loads.

The MC-DIC system also visually records the crack propagation process on beam surface, as shown in Figure 14. The cracks first appear in the mid-span as well as near the loading points. Combined with the phenomena observed in experiment, the main cracks often develop from cracks perpendicular to the bottom surface in the initial pure bending region. With the development of main cracks, there are often oblique cracks accompanying them. During the loading, the cracks continue to develop upward, being gradually intensified.

**Figure 15:**
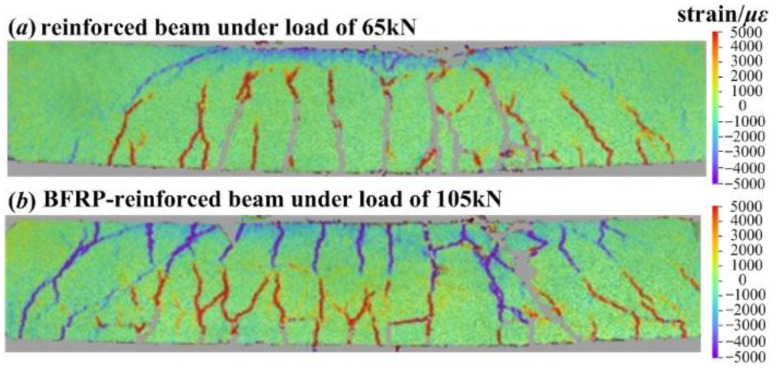
Comparison of crack propagations on (**a**) reinforced coral aggregate concrete beam under load of 65 kN and (**b**) BFRP-reinforced coral aggregate concrete beam under load of 105 kN.

Figure 15 presents the crack distributions on reinforced and BFRP-reinforced coral aggregate beams before the complete failure and spalling of concrete in compression zone. BFRP-reinforced beam exhibits a higher ultimate load than that of reinforced beam. It can be easily observed that in comparison with reinforced beam, the crack distribution in tension zone of BFRP-reinforced beam is denser, the damage in compression zone is more obvious, and the overall development of cracks is more complete. Specifically, the latter has more branch cracks and smaller crack spacing in the pure bending region, the root-like distribution characteristics of cracks are more obvious, and the crack development at the supports is more complete. In general, as opposed to the conventional manual depiction of cracks, the crack development obtained by MC-DIC is able to present the characteristics of cracks in a more intuitive manner.

## 4. Axial Compression Experiment of Timber Column

In this section, the circular timber column is taken as an example of the cylindrical civil components, the continuous measurement over the entire 360° cylindrical surface is performed using a continuous-view MC-DIC system. The 3D shape of the column is reconstructed, and the full-circle deformation evolution is measured. Moreover, the MC-DIC results are compared with the measurement data of strain gauge.

### 4.1. Experimental Program

For slender columns under compression, failure due to buckling instability will occur suddenly and lead to the structure collapse. Furthermore, due to the axisymmetric properties of circular columns, the direction of buckling instability is uncertain [50]. It is difficult to capture the true deflection direction and accurate buckling deformation via ordinary contact-type single-point electrical measurement methods, namely strain gauges, displacement transducers, etc. However, DIC can provide the non-contact full-field deformation, and specially MC-DIC can cover the entire cylindrical surface to obtain the continuous 360° full-circle deformation.

The axial compression experiments of several circular timber columns made of China fir are conducted to investigate the buckling instability of timber column subjected to compression. Note that only one specimen is employed here as an example to verify the effectiveness of continuous-view MC-DIC in deformation measurement of civil components with large curvature. More studies on stability are carried out in the literature [51].

**Figure 16:**
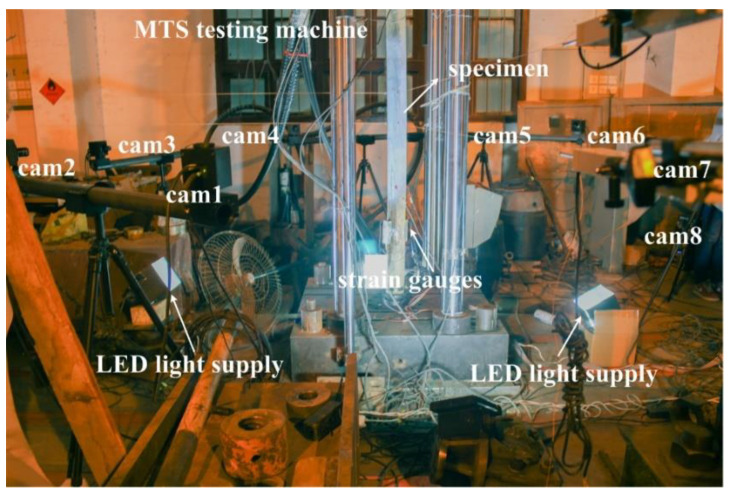
Experimental setup for the axial compression experiment of timber column.

The section diameter and length of example column is 100 mm and 1800 mm, respectively. A thin coating of random, black-and-white speckle particles is sprayed on column surface to create the speckle pattern required for DIC. The axial compression experiment of circular column with hinged ends is conducted with the MTS testing machine. Displacement control loading is adopted with the loading speed of 0.3 mm/min. The experimental setup is shown in Figure 16, containing eight industrial cameras (Point Grey GRAS-50S5M-C) with resolution of 2448 × 2048 pixels and pixel size of 3.45 μm, eight Kowa lenses with focal length of 25 mm, and LED light supplies.

**Figure 17:**
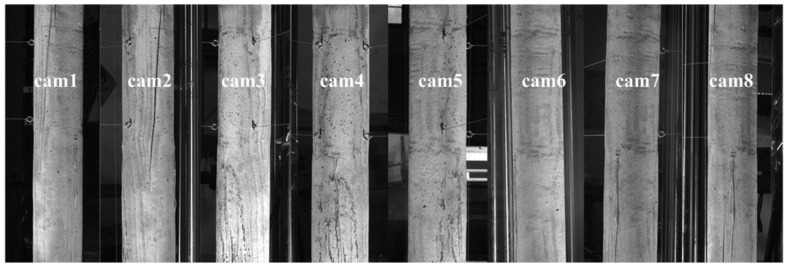
Local region captured by each camera.

The system construction process is as follows: (1) Instead of measuring the whole column length, only the mid-span part with larger deformation is taken for DIC measurement, that is the area from 300 mm above the mid-span to 300 mm below the mid-span. Therefore, the FOV height of DIC is set to 700 mm. (2) After determining the relative distance between cameras and specimen according to the measurement range, eight cameras are circumferentially arranged around the column with FOV of about 700 mm × 700 mm for each camera. The FOVs of adjacent cameras overlap by more than half, especially, the FOVs of camera 1 and camera 8 also need to be overlapped more than half, so that the end-to-end closed loop can be achieved. (3) Eight cameras are mounted rigidly to the high-strength carbon fiber tube through connecting sleeves, and then the tubes are fixed on six tripods, forming an eight-camera MC-DIC system. In the experiment, the exposure time of each camera is fixed to 25 ms and the aperture of each lens is set to moderate. For the given aperture and exposure time, sufficient contrast is obtained while avoiding the reflection and limiting motion blur, by adjusting the LCE light supplies. Eight cameras are synchronously controlled to capture images with acquisition frame rate of 1 frame/s. The local region captured by each camera is presented in Figure 17. Simultaneously, strain gauges are installed for the comparison between optical and electrical measurements.

### 4.2. DIC Results and Discussion

**Figure 18:**
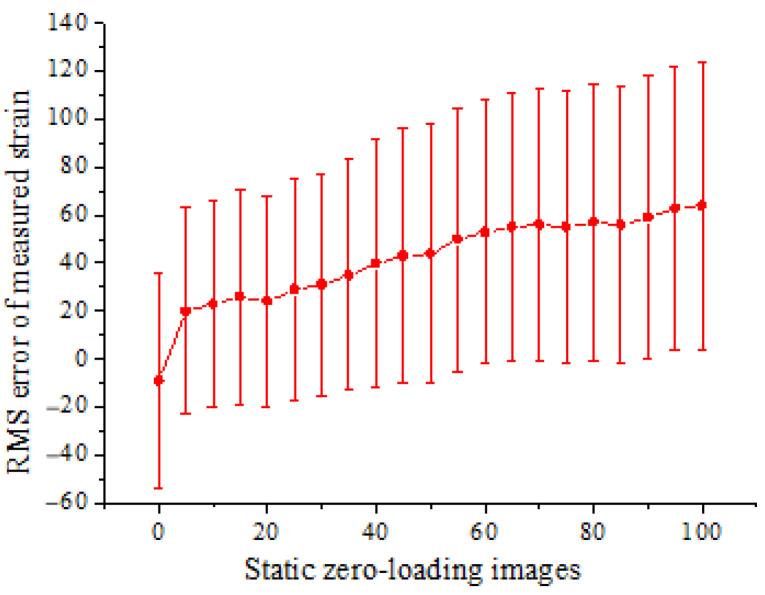
RMS error of strain computed from the static undeformed column.

To complete the noise analysis, a series of static images are taken from the undeformed specimen prior to the experiment. As shown in Figure 18, the measured strain is within ±130 με and shows an overall slight increasing trend.

**Figure 19:**
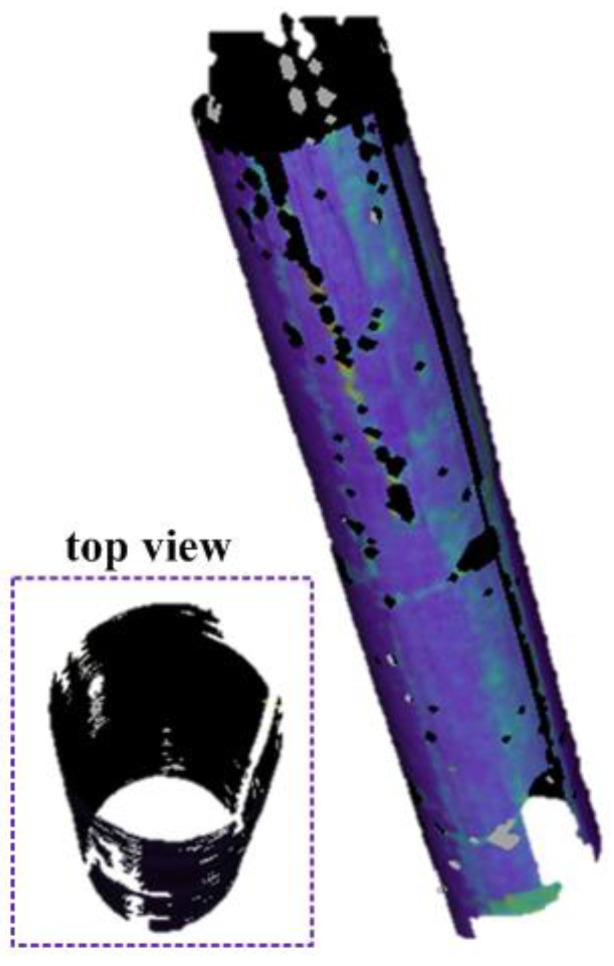
3D shape of the circular column [51].

For DIC calculation, the subset size is set to 29 × 29 pixels, the grid step size is set to 7 pixels, and the strain calculation window size is set to 15 × 15 calculation points. All the DIC parameters are selected considering the balance between smoothness and accuracy. As shown in Figure 19, MC-DIC is capable of fully reconstructing the 3D shape of the circular column. It should be pointed out that the missing parts on the following deformation fields are caused by the occlusion of strain gauges and connecting wires.

**Figure 20:**
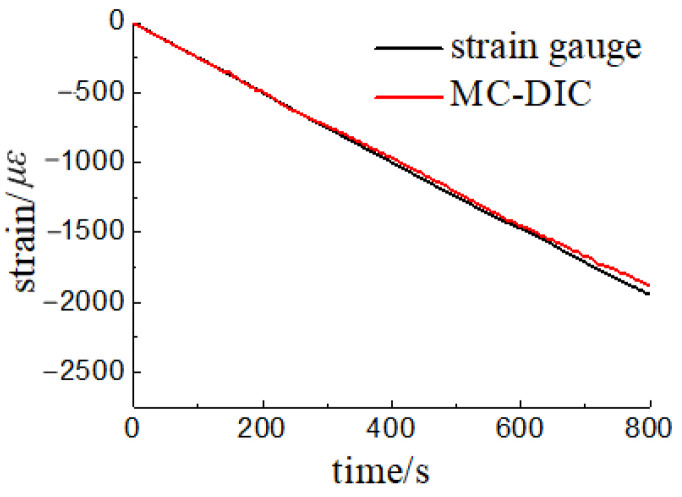
Results comparison of MC-DIC and strain gauge.

Similarly, we compare the strain gauge data with MC-DIC to verify the correctness of MC-DIC in strain measurement. It can be seen from Figure 20 that the data of strain gauge and MC-DIC are in good agreement with a maximum error of less than 3.8%. The difference between the two values tends to increase with the loading progress, which can be partially attributed to the fact that spray painting roughens the timber surface with loading time, resulting in a certain loss of accuracy in data extraction. This problem can be addressed by directly spraying black speckles on the specimen without a base coat of white paint or changing the speckle fabrication method, such as different transfer printing techniques, which deserves further experimental attempts.

**Figure 21:**
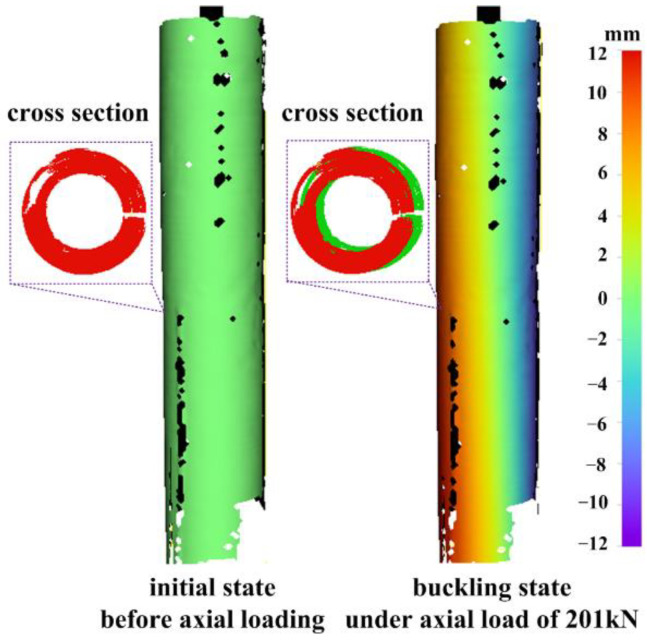
Full-circle lateral displacement field of the circular column under axial load.

The lateral displacement field, reflecting the buckling direction, is depicted in Figure 21. From the perspective of cross section, green circle denotes the initial section, red circle represents the current section, and red circle is placed on top of green circle to reveal the lateral displacement. In the initial state before axial loading, the current section is exactly the initial section, so red and green circles overlap. In the buckling state under axial load of 201 kN, the current section (red) is offset compared to the initial section (green), indicating that a lateral displacement has occurred. Observed from the displacement field, there is a convex displacement on one side of the column (orange part to the left) and a concave displacement on the other side (blue part to the right).

**Figure 22:**
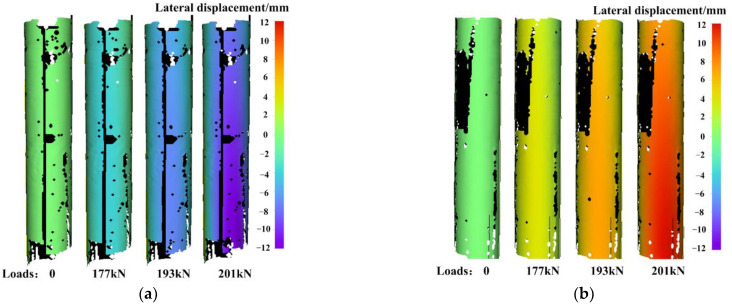
Displacement evolution on: (**a**) concave side; (**b**) convex side under different axial loads.

To more clearly illustrate the deformation evolution on entire 360° cylindrical surface, Figure 22a,b presents the displacement evolution on the concave and convex sides of the column under different axial loads, respectively. The uncalculated black parts on the deformation fields are caused by occlusion of strain gauges and connecting wires. As the loading progresses, the displacements on the concave and convex sides gradually increase from 0 to about 12 mm.

**Figure 23:**
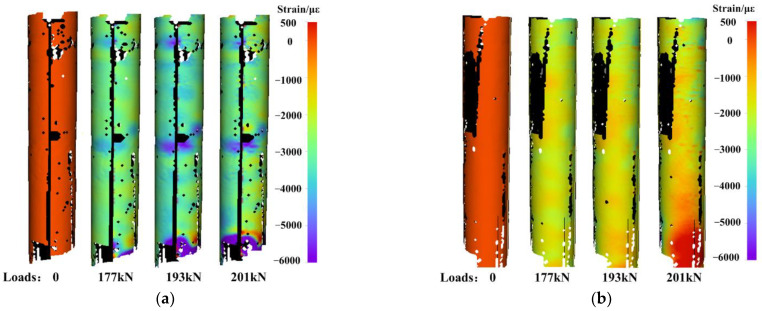
Strain evolution on: (**a**) concave side; (**b**) convex side under different axial loads.

Correspondingly, MC-DIC can also obtain the full-circle strain field, the strain evolution on the concave and convex sides under different axial loads are shown in Figure 23a,b, respectively. First, the compressive strain on the column surface gradually increases with the loading. Secondly, the compressive strain around the column is not evenly distributed, the concave side undergoes a larger compressive strain than the convex side. The concave surface finally has a strain value up to about 5000 με, while the convex surface has a maximum strain value of around 2500 με. It should be noted that the data with larger errors at the lower end of the column are not taken into consideration. During the measurement, to avoid the speckles in AOI from being arbitrarily blocked, all the dangling wires of strain gauges are gathered together along a line (see in Figure 16 and Figure 17) and concentrated to the lower end. Besides, the speckle range is larger than the AOI to ensure enough valid data. Thus, the speckle spraying quality at the edges away from the mid-span is relatively poor. All above results in the local large-error area in the lower edge.

## 5. Conclusions

The most common civil components are the beam as flexural member and column as vertical load-bearing member. To address the limitations of conventional stereo-DIC in measuring these components, a continuous-view MC-DIC system with two forms of camera arrangement is developed. Two experiments of slender beam and circular column are taken for instance to verify the capability of continuous-view MC-DIC in the deformation measurement of civil components with slenderness ratio and large curvature.

1. In the bending experiment of coral aggregate concrete beam, the full-surface deformation distribution and crack propagation are visually recorded. The data obtained by MC-DIC are in good agreement with displacement transducer, and the vertical displacement field is consistent with the deformation characteristics of the four-point bending beam. Moreover, as opposed to the conventional manual depiction of cracks, the crack propagation obtained by MC-DIC is able to present the characteristics of cracks in a more intuitive manner.

2. For the axial compression experiment of timber column, the 3D shape of the circular column is fully reconstructed, and the full-circle lateral displacement field, reflecting the buckling direction, is also obtained. The compressive strain around the column is not evenly distributed, and the concave side undergoes a larger compressive strain than the convex side. Moreover, the strain estimated by MC-DIC maintains good consistency with the strain gauge.

3. Results demonstrate that the continuous-view MC-DIC is capable of realizing the continuous full-surface measurement of civil components with large slenderness ratio and with large curvature. The continuous-view MC-DIC is proven to be a reliable 3D full-field measurement approach in civil measurements.

## Data Availability

The data that support the findings of this study are available upon reasonable request from the authors.
